# Structural Racism and Long-term Disparities in Youth Exposure to Firearm Violence

**DOI:** 10.1001/jamanetworkopen.2023.12425

**Published:** 2023-05-01

**Authors:** Jonathan Jay

**Affiliations:** Department of Community Health Sciences, Boston University School of Public Health, Boston, Massachusetts.

Young people’s exposure to firearm violence in US cities has surged during the COVID-19 pandemic.^1,2^ This study by Lanfear and colleagues^3^ examines how long-term changes in firearm violence have influenced exposure across multiple birth cohorts in Chicago, Illinois. Comparing 4 cohorts from the Project on Human Development in Chicago Neighborhoods (PHDCN), Lanfear et al^3^ find that any benefits younger cohorts might have from declines in citywide violence from 1994 to 2014 were erased by subsequent violence increases. The results underscore how little progress we have made over several decades to address the root causes of community firearm violence and its disproportionate effects on Black and Hispanic youth.

In the study by Lanfear and colleagues,^3^ the youngest PHDCN cohort, born around 1996, entered adulthood after a sustained lull in firearm homicide rates in Chicago (ie, 2004-2014). This lull limited the cohort’s exposure to neighborhood experiences of firearm violence, such as seeing someone get shot, during early life. However, these youth were in the highest-risk phase of adolescence (ages 18-24 years) when firearm violence spiked in 2016 and again in 2020.^3^ Consequently, their cumulative risk of being shot was comparable to cohorts born earlier, including those who entered adolescence during the most violent years of the 1990s.^3^

Exposure to firearm violence is associated with lasting consequences for youth and their loved ones. Indirect exposure (eg, witnessing violence) and direct exposure (eg, surviving an assault) can influence mental and physical health outcomes over the life course. In a subset of individuals, exposure is associated with the future enactment of firearm violence, feeding cycles of firearm violence at the community level. The increase in firearm violence during the COVID-19 pandemic is likely to have long-term outcomes because it substantially increased both indirect^1^ and direct^2^ exposure among US youth. Therefore, it is crucial to continue scaling up public health programs to halt the violence surge and deal with its aftermath, especially through community-based outreach programs and trauma-informed services.

At the same time, efforts must directly target the systemic inequities that concentrate firearm violence exposure among Black and Hispanic youth. Racial and ethnic disparities in these outcomes are profound and longstanding. In the study by Lanfear et al,^3^ differences in firearm violence exposure by race were more extreme than differences between birth cohorts. By the end of the study period, exposure was roughly comparable across age cohorts but exposure was consistently lowest among White youth. Other recent work, such as a 2023 study by Jay et al,^2^ reported that during the pandemic, Black children were 100 times, and Hispanic children 25 times, more likely than White children to be shot in Chicago and other major US cities.

These staggering disparities demand action against structural racism as a driver of firearm violence. Incremental violence reductions, such as those experienced by many US cities from the mid-1990s through mid-2010s, are necessary and life-saving but also precarious, as recent spikes have shown. There is no consensus among scholars on the reasons for the long-term declines in violence, but it is evident that these declines did not fundamentally disrupt the racial inequities that determine who is at the most risk of being shot or witnessing a shooting: even as homicide rates declined in Chicago, violence was increasingly concentrated in predominantly Black neighborhoods.^4^

Although community violence had been gradually declining, Black and Hispanic youth remained pervasively exposed to societal inequities. Child poverty in the US peaked in the early 2010s, with more than one-quarter of Black and Hispanic children living below the federal poverty line.^5^ Meanwhile, policy makers emphasized punitive responses, such as aggressive policing and harsh sentencing, even for nonviolent offenses. Black and Hispanic communities bore the brunt of these policies. In Chicago, for example, mass incarceration “hot spots” emerged, mainly in Black neighborhoods on the South and West sides. There were 851 “million dollar blocks,” where the costs of incarcerating residents from these areas exceeded $1 million, from 2005 to 2009 alone.^6^ Such massive investments in punishment necessarily came at the expense of community investments that might have helped close racial and ethnic gaps in child opportunity.

This disinvestment from basic needs likely explains why Black and Hispanic youth experienced the largest change in violence exposure during the COVID-19 pandemic. When violence increased during the pandemic, it increased fastest in the neighborhoods most burdened by racial segregation and economic deprivation,^7^ where COVID-19 deaths, economic losses, and other stressors were most severe. Firearm violence exposure among White youth mostly remained low during the pandemic, suggesting that the 2020 spike was not inevitable, but rather a byproduct of the social and economic vulnerability produced by sociopolitical forces, such as long-term disinvestment.

Addressing racial and ethnic disparities in youth exposure to firearm violence requires not only interrupting cycles of violence, but interrupting cycles of racialized disinvestment and punitive policy-making. Community investments at the micro level, such as improving the physical condition of abandoned properties, have been shown to reduce nearby firearm violence^8^; at the macro level, the federal Child Tax Credit caused child poverty to plummet in 2021.^5^ Policies to reduce residential racial segregation, such as inclusionary zoning, are also likely necessary to close gaps in the long term. There was not much change in firearm violence exposure for youth born between the early 1980s and late 1990s in Chicago—and until we remove the structural barriers to community safety, this change is unlikely to come.
